# Childhood Neurodevelopmental Outcome in Low Birth Weight Infants With Post-ligation Cardiac Syndrome After Ductus Arteriosus Closure

**DOI:** 10.3389/fphys.2019.00718

**Published:** 2019-06-11

**Authors:** Maria Carmen Bravo, Marta Ybarra, Rosario Madero, Adelina Pellicer

**Affiliations:** ^1^Department of Neonatology, La Paz University Hospital, Madrid, Spain; ^2^Division of Statistics, La Paz University Hospital, Madrid, Spain

**Keywords:** ductus arteriosus, preterm, cardiorespiratory instability, ductus ligation, neurodevelopment

## Abstract

**Background:**

Post-ligation cardiac syndrome (PLCS) is a common complication of patent ductus arteriosus (PDA) surgical closure in low birth weight infants. It has been associated with mortality, but there is a lack of information about the neurodevelopmental outcome of the survivors. We aimed to explore the prevalence of PLCS and to assess whether this clinical condition is a risk factor for adverse outcome, (moderate or severe neurodevelopmental disabilities).

**Methods:**

We retrospectively reviewed the medical charts of all the infants < 30 weeks of gestation who underwent ductus arteriosus ligation at our unit between 2005 and 2009.

**Results:**

During the study period, 39 preterm infants [mean gestational age 26.4 (2) weeks] underwent surgical closure of the PDA at a mean postnatal age of 25.3 (2.3) days. Twenty six percent of the study population developed PLCS. Five infants died and the follow-up was accomplished in 24 infants (70% of the survivors) at a mean age of 5.3 (1.5) years (range 2–9 years). Neurodevelopmental impairment was observed in 6 in the PLCS group (75%) and in 6 infants in the no PLCS group (37%), *p* = 0.08]. Multiple regression analyses showed that the best fitting model for predicting adverse outcome included PLCS and low birth weight, *p* = 0.018.

**Conclusion:**

Preterm infants undergoing surgical closure of PDA who fulfill the criteria of PLCS according to this study seem to have a tendency toward higher risk of long-term neurodevelopmental impairment. Prospective clinical trials reporting long-term follow-up data should be designed to confirm the hypotheses generated in this pilot study.

## Introduction

Post-ligation cardiac syndrome (PLCS), characterized by hypotension requiring cardiovascular support and ventilation or oxygenation failure, complicates the postoperative course of the surgically closed patent ductus arteriosus (PDA). The condition is particularly common in the youngest and most immature infants (McNamara et al., 2010; Sehgal et al., 2010), or in those who required cardiovascular support before surgery (Teixeira et al., 2008; Sehgal et al., 2010). Compromised ventricular performance as a result of increased systemic vascular resistance in combination with a decrease in left ventricular preload, are thought to underlie the pathophysiology of PLCS (McNamara et al., 2010; El-Khuffash et al., 2013, 2014). Increased mortality associated with PLCS has been shown (Harting et al., 2008). An increased risk of neurosensory impairment delay has been reported in infants who underwent PDA surgical closure (Kabra et al., 2007; Weisz et al., 2014). However, the primary determinants of adverse outcomes in those who undergo surgical PDA closure are confounded by the indication for this particular surgery that may be associated with adverse outcome.

We hypothesize that PLCS itself is a risk factor for impaired neurodevelopmental outcome. Therefore, we conducted a retrospective review in a cohort of infants who underwent surgical closure of the PDA. Our primary aim was to assess the prevalence of PLCS and to evaluate the impact of this condition on the long-term neurodevelopmental outcome.

## Materials and Methods

Clinical charts were reviewed of all infants born younger than 30 weeks of gestation who were free of congenital heart disease, and who underwent PDA ligation at the La Paz University Hospital in Madrid, Spain, between January 2005 and November 2009. The study was approved by the hospital’s Ethics Committee. All the infants underwent a muscle-sparing posterolateral thoracotomy and clip occlusion. Surgery was conducted at the NICU in all cases. Anesthetic management was standardized using total intravenous anesthesia with fentanyle and neuromuscular blockade with cisatracurium. Changes in ventilation parameters or cardiovascular support were done at the discretion of the attending clinician. Cardiovascular management after surgery was guided by a constellation of routine clinical or biochemical data, mainly blood pressure, following a standardized protocol: dopamine (first line), epinephrine (second line), and dobutamine (third line) plus/minus volume expansion; and hydrocortisone in case of refractory shock. The following perinatal variables potentially influencing neonatal outcomes were considered: gestational age, birth weight, sex, antenatal steroids, cord pH, Apgar score, need for advanced resuscitation at birth, and cesarean section. Pre-surgery clinical and echocardiographic features of pulmonary over-circulation and systemic hypoperfusion that supported the indication of PDA ligation, and the postnatal age at surgery were also recorded. The severity of ductal disease before surgery was retrospectively classified by one of the study researchers (MCB) using the Teixeira’s grading system (Teixeira et al., 2008). Accordingly, category I included infants with profuse pulmonary hemorrhage or cardiocirculatory failure requiring > 2 inotropes; category II, included babies with deteriorating respiratory status, preterm babies < 26 weeks of gestation with large PDA and clinical contraindication for medical treatment, cardiocirculatory failure requiring > 1 inotropes, or necrotizing enterocolitis and a large PDA; and finally, category III was reserved for inability to extubate or wean respiratory support or cardiocirculatory failure associated with failure to thrive (Teixeira et al., 2008). The pre- and immediate postoperative (24-h period after PDA ligation) respiratory and circulatory status were systematically evaluated including the following data: type of ventilator support, supplemental oxygen, blood gasses and acid-base status, blood pressure, heart rate, and inotrope score (IS) [(dopamine dose × 1) + (dobutamine dose × 1) + (epinephrine dose × 10) in μg/kg/min] (Wernovsky et al., 1995). PLCS was defined as the need for cardiovascular support escalation, and either oxygenation or ventilation failure that were unexplained by other surgery-related complications. Cardiovascular support escalation was considered in the case of more than 20% increase in IS or need for fluid bolus administration (Jain et al., 2012). Oxygenation failure was considered when a non-transient (longer than 1 h) ≥ 20% increase in the inspired oxygen fraction or mean airway pressure was observed. Finally, ventilation failure was defined as need to switching to high frequency oscillatory ventilation or a consistent (longer than 1 h) ≥ 20% amplitude increase if already on high frequency oscillatory ventilation. Other surgery-related complications such as pneumothorax, vocal cord palsy, surgical wound infection, or immediate postoperative mortality were recorded. The main neonatal clinical outcomes were documented, that included the most severe cranial ultrasound diagnoses made by an expert neonatologist on brain ultrasonography who was blind to which group the patients belonged [(intraventricular hemorrhage (IVH), periventricular haemorrhagic infarction (PVHI), or white matter damage (WMD) at term equivalence)]. WMD was defined as porencephalic cysts, cystic periventricular leukomalacia, cerebral atrophy or persistent periventricular echogenicity (PVE) at term age.

### Follow-Up

The surviving infants were included in the routine follow-up program and underwent regular physical and motor exams by an experienced team of neonatologist, psychologist, pediatric neurologist and pediatric psychiatrists if needed that were blinded to whether the infants had PLCS or not. Neurodevelopment was evaluated by the Bayley scales of infants development, second edition (BSID-II) (Bayley, 1993) at 2 years’ corrected age. Test results were adjusted for prematurity. Cognitive assessment was performed in children aged 4–6 years using the Wechsler preschool and primary scale of intelligence (WPSI), Third Edition (Wechsler, 2010) or Kaufman Brief Intelligence Test, Second Edition (KBIT-2) (Kaufman and Kaufman, 2011). The history of vision, hearing function and behavioral problems was collected from the medical records. Cerebral palsy (CP) was scored according to the Gross Motor Function Classification System (Cans, 2000). Neurodevelopmental disabilities were classified as: (1) absent or mild (no CP or GMFCS I; Bayley II mental and psychomotor scores > 84, global intelligence quotient, IQ, >70; normal hearing and normal vision); (2) moderate or severe (GMFCS level ≥ II; or Bayley II scores ≤ 84; or global IQ ≤ 70; or sensorineural hearing loss defined as a hearing threshold ≥ 20 dB by auditory brainstem responses; or visual deficit; or behavioral/emotional problems including affective problems, anxiety, somatic complaints, attention deficit/hyperactivity, oppositional defiant or rule-breaking behavior or pervasive developmental disorder (Alarcon et al., 2016).

The data were analyzed using the statistical software SPSS for windows, version 25. The quantitative data are expressed as means (standard deviation) and qualitative data as counts (percentages). The perinatal data, perioperative variables, and outcome variables of the study population (PLCS vs. no PLCS groups) were compared using the Mann-Whitney *U* test and Fisher’s exact test. There were also studied, using a multiple regression analyses, the perinatal variables that could be associated with moderate or severe neurodevelopmental disabilities in order to create the best model for predicting adverse outcome. All the statistical analyses were considered bilateral, and values of *p* < 0.05 were considered significant.

## Results

Forty-two infants underwent surgical closure of the PDA during the investigation period. The clinical charts of 3 infants were poor or incomplete; thus, only 39 preterm infants with a mean gestational age of 26.4 (2) weeks formed the studied cohort. The surgery lasted a median of 45 min with a range of 25–140 min. The perinatal variables between the 10 infants who developed PLCS (26%, PLCS group) and the 29 infants who did not develop the condition (74%, no PLCS group) did not differ (Table 1). Eighty-seven per cent (*n* = 34) of the study population received medical treatment for PDA closure before surgery (17 indomethacin, 14 ibuprofen, and 3 both medications). Thus, for 5 infants (13% of the cohort) surgery was primarily indicated due to a contraindication for medical treatment (renal insufficiency, severe IVH, necrotising enterocolitis, or bowel perforation). PDA category I before surgery was more common in the PLCS group, whereas those in the no PLCS group were more frequently classified as PDA category III (Table 1). Before surgery, infants in the PLCS group had higher mean airway pressure than those in the no PLCS group, although not statistically significant [PLCS group 9.4 (2.3) cm H_2_O; no PLCS group 7.7 (2) cm H_2_O; *p* = 0.06]. No other haemodynamic or respiratory variables differed between the study groups before surgery. The evolution of the IS after surgery is shown in Table 1. All the infants in the PLCS group and 11 (38%) infants in the no PLCS group developed oxygenation failure (*p* = 0.002); one infant in the PLCS group also had ventilation failure. Other surgery-related complications were pneumothorax (*n* = 2, 5%) and vocal cord palsy (*n* = 5, 13%), without differences between study groups. No immediate postoperative mortality or surgical wound infection were found in this series.

**Table 1:** Perinatal, perioperative, and outcome variables.

	PLCS group (*n* = 10)	No PLCS group (*n* = 29)	p
**Perinatal and pre-ligation variables**			
Gestational age, wks (SD)	25.6 (1.8)	26.6 (1.9)	0.1
Birth weight, g (SD)	822 (124)	884 (253)	0.3
SGA, n (%)	0 (0)	3 (10)	0.5
Male, n (%)	7 (70)	20 (69)	1
Multiple birth, n (%)	4 (40)	10 (34)	1
Antenatal steroids, n (%)	6 (60)	17 (59)	0.9
Cesarean section, n (%)	6 (60)	20 (69)	0.7
Advanced resuscitation, n (%)	8 (80)	21 (72)	1
Apgar 5 min (SD)	7.2 (1.2)	6.9 (1.2)	0.5
Cord pH (SD)	7.27 (1.4)	7.31 (0.04)	0.5
HMD, n (%)	10 (100)	25 (86)	0.5
IVH grade III or PVHI, n (%)	1 (10)	4 (14)	1
**Perioperative variables**			
PDA medical treatment, n (%)	9 (90)	24 (83)	1
Postnatal age at surgery, days (SD)	22 (12)	26 (25)	0.4
Ductus diameter, mm (SD)	2.5 (0.7)	2.6 (1)	0.8
Ductus category I, n (%)	4 (40)	3 (10)	0.06
Ductus category II, n (%)	5 (50)	10 (34)	0.4
Ductus category III, n (%)	1 (10)	15 (52)	0.03
IS before surgery (SD)	10.2 (12)	3.6 (5)	0.1
IS 1 h after surgery (SD)	11.8 (14)	3.9 (5.7)	0.1
IS 8 h after surgery (SD)	13 (10.7)	3.2 (5.2)	0.02
IS 12 h after surgery (SD)	12.3 (12.3)	2.8 (5)	0.052
IS 24 h after surgery (SD)	16 (13.4)	1.5 (3)	0.008
**Outcome variables at term equivalence**			
Mortality, n (%)	1 (10)	4 (14)	1
ROP requiring laser therapy, n (%)	5 (50)	10 (34)	0.2
Necrotizing enterocolitis, n (%)	3 (30)	7 (24)	0.7
BPD, n (%)	6 (85)	14 (64)	0.4
WMD, n (%)	7 (70)	14 (52)	0.5

*Mann-Whitney U test and Fisher’s exact test. Quantitative data are presented as means (SD) and qualitative data as count (percentages). SGA, small for gestational age; antenatal steroids, complete course; advanced resuscitation, endotracheal intubation in the delivery room; HMD, hyaline membrane disease; IS, inotropic score (Wernovsky et al., 1995) [(dopamine dose × 1) + (dobutamine dose × 1) + (epinephrine dose × 10)] ROP, retinopathy of prematurity; BPD, bronchopulmonary dysplasia defined as supplemental oxygen at 36 weeks of gestation; IVH, intraventricular hemorrhage; PVHI, periventricular haemorrhagic infarction; WMD, white matter damage.*

Five infants (13%) died, without differences between groups. Among survivors, 70% (*n* = 24) were followed until a mean age of 5.3 (1.5) years (range 2–9 years). Patients lost to follow-up were primarily due to transfer to the referral hospital after PDA ligation [*n* = 1 (10%) in the PLCS group; and *n* = 9 (31%) in the no PLCS group; *p* = 0.3]. No differences in the perinatal or pre-ligation variables were observed between the infants that were followed and those who were not (gestational age, 26.4 (1.8) and 26.3 (2.2) weeks, *p* = 0.9; birth weight, 870 (210) and 864 (284) g, *p* = 0.9; PDA category I, *n* = 5 (17.2%) and *n* = 2 (20%), *p* = 0.8). The main neonatal clinical outcomes at term equivalence are shown in Table 1.

At follow-up, moderate or severe neurodevelopmental impairment was more prevalent in those infants who developed PLCS after ductus surgery [*n* = 6 in the PLCS group (75%) and in 6 infants in the no PLCS group (37%), *p* = 0.08. Low birth weight was also associated with neurodevelopmental disability (*p* = 0.013). We found that the cut-off value with 67% of sensitivity and specificity for neurodevelopmental impairment was 850 gr at birth, *p* = 0.1. Thus, the best fitting model for predicting moderate or severe neurodevelopmental disabilities included PLCS and birth weight, *p* = 0.018. Neither the gestational age nor the ductus category before surgery improved the predictive model (only one of the infants with neurodevelopmental disabilities was classified as ductus category I).

The follow-up of the infants with adverse outcome (death or any grade of moderate or severe neurodevelopmental impairment) is described in Table 2.

**Table 2:** Infants with adverse outcome defined as death or any grade of moderate or severe neurodevelopmental impairment.

Patient	GA (weeks)	Ductus category	PLCS	Most severe CUS diagnose	Death	Long-term outcome: findings	Follow up (years)
7	25.3	I	No	IVH grade II and WMD (PVE)	Yes		–
11	24	II	Yes	WMD (PVE)	No	MDI 75 at 2 years’ corrected age PDI 88 at 2 years’ corrected age SNHL CP (GMFCS level III): spastic diplegia	5
13	24.7	II	No	WMD (cerebral atrophy and PVE)	No	MDI 93 at 2 years’ corrected age PDI 83 at 2 years’ corrected age Motor and language delay at 5 years old	5
12	24	II	Yes	WMD and IVH grade II	No	Attention deficit/hyperactivity Language delay at 5 years old	5
14	27.6	II	Yes	IVH grade II	No	MDI 90 at 2 years’ corrected age PDI 80 at 2 years’ corrected age CP (GMFCS level I): spastic diplegia Attention deficit/hyperactivity	4
17	24	I	Yes	WMD (cerebral atrophy and PVE)	No	SNHL and visual deficit Autism spectrum disorder Motor, cognitive and language delay at 8 years old	8
19	26.7	I	No	IVH grade III and PHH	Yes		–
23	24.1	III	No	IVH grade II and WMD (PVE)	No	MDI 91 at 2 years’ corrected age PDI 83 at 2 years’ corrected age Motor delay at 5 years old	5
24	25.4	III	No	IVH grade II and WMD	No	MDI 71 at 2 years’ corrected age PDI 58 at 2 years’ corrected age Motor and language delay at 6 years old	6
25	26	III	No	WMD (PVE)	No	MDI 95 at 2 years’ corrected age PDI 80 at 2 years’ corrected age	2
28	27.1	III	No	IVH grade III and PVHI	Yes		–
31	30.3	II	No	WMD (PVE)	Yes		–
32	28.6	III	No	Normal	No	Attention deficit/hyperactivity CI 73 Motor and language delay at 6.5 years old	6.5
34	24.2	I	Yes	IVH grade II	Yes		–
36	26.1	II	Yes	IVH grade II	No	MDI 83 at 2 years’ corrected age PDI 89 at 2 years’ corrected age Rule-breaking behavior	5
37	26	III	Yes	WMD (PVE)	No	Motor and language delay Attention deficit/hyperactivity Rule-breaking behavior	7
40	24.7	III	No	IVH grade II and WMD (PVE)	No	MDI 72 at 2 years’ corrected age PDI 79 at 2 years’ corrected age Language delay at 5 years old	5

*GA, gestational age; PLCS, postligation cardiac syndrome; CUS, cranial ultrasound; WMD, white matter damage; PVE, persistent periventricular echogenicity at term age; IVH, intraventricular hemorrhage; PVHI, periventricular haemorrhagic infarction; posthaemorrhagic hydrocephalus; MDI, Bayley scale II-mental developmental index; PMI, Bayley scale II-psychomotor developmental index; SNHL, sensorineural hearing loss; CP, cerebral palsy; GMFCS, gross motor function classification system; WPSI, Wechsler preschool and primary scale of intelligence.*

## Discussion

This is the first study reporting on the impact that PLCS itself has on the long-term outcome of preterm infants undergoing PDA surgical closure. PLCS has been associated with mortality (Harting et al., 2008) and we have shown that the survivors who developed this condition after surgery seem to be at higher risk of neurodevelopmental impairment, although we could not find a statistical association probably due to the small sample size of this study. These findings have two implications. First, surgical ligation of the PDA could be a risk factor for neurodevelopmental impairment as the association between this kind of treatment and poor neurologic outcome has been reported (Kabra et al., 2007; Weisz et al., 2014). However, most infants undergoing surgery are also the sickest; thus, in the absence of randomized clinical trials addressing the causal role of the surgical procedure on the adverse outcome, it is essential to further characterize which infants undergoing PDA ligation are exposed to an increased risk for neurodevelopmental impairment. This step is crucial, given that surgery is considered a rescue treatment in the case of medical treatment failure or contraindication for cyclooxygenase inhibitor prescription. In this report we have observed that infants with profuse pulmonary hemorrhage or cardiocirculatory failure requiring > 2 inotropes before surgery are at higher risk of developing PLCS. Although PLCS is commonly observed in the sickest infants, we hypothesize whether PLCS, especially when is observed in the infants with a birth weight below 850 gr, could be an independent risk factor for neurodevelopmental impairment (neither the gestational age nor the ductus category before surgery were associated with poor neurodevelopmental outcome). Second, the primary determinants for the development of PLCS are the combination of impaired myocardial performance together with increased systemic vascular resistance and a sudden reduction in preload (Teixeira et al., 2008; McNamara et al., 2010; Jain et al., 2012; Ting et al., 2016). Regrettably, this retrospective study did not systematically evaluate cardiac performance by echocardiography; however, the escalation in cardiovascular treatment that was observed during the immediate postoperative period supports this notion. In fact, our patients clearly shown differential IS after surgery that was statistically significant across the whole postoperative observation period. To date, milrinone has been proposed as a preventive intervention in this population (Jain et al., 2012; Ting et al., 2016). Randomized clinical trials to address the efficacy and safety of this inodilator in this indication would be of utmost interest. It is, therefore, essential to fully characterize which infants are at greater risk of PLCS in the face of implementing preventive or treatment strategies.

Our study and previous reports (Harting et al., 2008) suggest that PLCS occurs in one in 4 or 5 preterm infants who undergo PDA surgical closure. PLCS has been associated with immaturity (Harting et al., 2008; McNamara et al., 2010; Sehgal et al., 2010) and greater need for cardiovascular support before surgery (Harting et al., 2008; Teixeira et al., 2008; Sehgal et al., 2010). We did not observe differences in either the perinatal or the perioperative variables among those who developed PLCS and those who did not, probably due to the small sample size. However, the infants in this series who suffered PLCS had a PDA that was categorized as being more severe than that of the infants without PLCS.

After analyzing the potential confounders for predicting neurodevelopmental impairment, we observed that, as the low birth weight was the only perinatal risk factor associated with adverse outcome in this study, the best predicted model for moderate or severe neurodevelopmental disability included PLCS and birth weight < 850 gr.

There are several limitations to our study. First of all, the small sample size could explain the lack of association between the gestational age and the neurodevelopmental impairment. Secondly, by the time this cohort underwent the surgery, the cardiovascular management was guided by a constellation of routine clinical or biochemical data, mainly blood pressure. Now, in our unit, the cardiovascular support after ductus surgery follows a pathophysiological approach as published recently (El-Khuffash et al., 2014), being also guided by functional echocardiography. Furthermore, this is a retrospective study with a small sample size, and a proportion of patients were lost to follow-up. However, the followed cohort appropriately represents the entire cohort, given the patients who were lost to follow-up are balanced between the two groups (PLCS group and no PLCS group). In addition, it is difficult to comply with a full follow-up program when the standard of care consists on transfer back to the referral hospital after PDA ligation in uncomplicated surgery. For this reason, the follow-up up to the mean age of 5.3 years in this cohort is a strength of the study.

In summary, preterm infants undergoing surgical PDA closure that fulfill the criteria of PLCS according to this study seem to have a tendency toward higher risk of moderate or severe long-term neurodevelopmental impairment, especially those with a birth weight below 850 gr. However, this is a pilot study, so prospective clinical trials reporting long-term follow-up data should be designed to confirm this data. Randomized clinical trials on preventive or treatment strategies for PLCS are also warranted.

## Ethics Statement

This study was approved by The Ethics Committee for Human Studies at La Paz University Hospital as it was performed in accordance with the ethical standards laid down in the 1964 Declaration of Helsinki and its later amendments. This Ethics Committee also concluded that it was not necessary to obtain an individual informed consent for all participants as this is a retrospective reviewed of the clinical charts of the infants born between 2005 and November 2009.

## Author Contributions

MB conceived and designed the study, drafted the initial manuscript, collected the information from the medical charts, performed the initial analyses, and approved the final manuscript as submitted. MY collected the information from the medical charts and approved the final manuscript as submitted. RM performed the statistical analysis, reviewed the manuscript and approved the final manuscript as submitted. AP conceived and designed the study, drafted the initial manuscript and approved the final manuscript as submitted.

## Conflict of Interest Statement

The authors declare that the research was conducted in the absence of any commercial or financial relationships that could be construed as a potential conflict of interest.

## Abbreviations

CP: cerebral palsy
IS: inotrope score
IVH: intraventricular hemorrhage
MPC: mental processing composite of the kaufman assessment battery for children
PDA: patent ductus arteriosus
PVHI: periventricular haemorrhagic infarction
PLCS: post-ligation cardiac syndrome
WMD: white matter damage.
